# Infection with *Nocardia pneumoniae* in a 54-year-old woman with bronchiectasis – a case report and review of the literature

**DOI:** 10.1016/j.rmcr.2025.102326

**Published:** 2025-11-20

**Authors:** Adrian Endres, Maria Pilot, Michael Hogardt, Sarah Westeppe, Birgit Zirn, Gernot Rohde

**Affiliations:** a Department of Pneumology and Allergology, Department of Internal Medicine I, University Hospital Frankfurt, 60329, Frankfurt am Main, Germany; b Department of Medicine, Pulmonary and Critical Care Medicine, University Medical Center Giessen and Marburg, Philipps University of Marburg, Marburg, 35043, Germany; c Institute for Medical Microbiology and Infection Control, University Hospital Frankfurt, Goethe University Frankfurt, 60329, Frankfurt am Main, Germany; d German National Consiliary Laboratory on Cystic Fibrosis Bacteriology, Frankfurt am Main, Germany; e Dr. Senckenberg Institute of Human Genetics, University Hospital and Goethe University, 60596, Frankfurt am Main, Germany

**Keywords:** Nocardia, Nocardia pneumoniae, Bronchiectasis, Case report

## Abstract

**Background/purpose:**

*Nocardia* spp. are rare but clinically relevant pathogens in patients with structural lung disease and/or immunosuppression. *Nocardia pneumoniae* was first described in 2004; however, its clinical relevance has remained unclear due to the limited number of reported cases to date.

**Methods:**

Case report and review of the literature.

**Case presentation:**

We report a case of a 54-year-old female with bronchiectasis who presented with pulmonary exacerbation and was diagnosed with an infection with *Nocardia pneumoniae*. The current case was successfully treated with trimethoprim-sulfamethoxazole monotherapy over 12 weeks.

**Conclusion:**

We propose that *Nocardia pneumoniae* should be considered clinically significant. The management of nocardiosis remains difficult, as reliable evidence on optimal antimicrobial strategies, appropriate treatment duration, and the need for secondary prophylaxis is still lacking.

## Highlights

• The case presented supports the role of *Nocardia pneumoniae* as a human pathogen.
• Successful TMP-SMX therapy.
• Review of all previously published clinical cases of *Nocardia pneumoniae* infection.

## 1 Introduction

The genus *Nocardia* belongs to the class *Actinobacteria* in the order *Corynebacteriales* [1], that are ubiquitously found in the environment. There are currently about 109 validly named *Nocardia* spp. with over 50 shown to be clinically significant [[2], [3], [4]]. *Nocardia* are typically opportunistic pathogens that more frequently affect immunocompromised patients [5,6]. Nocardiosis primarily affects the lung but may also cause skin and soft tissue infection, cerebral abscess, bloodstream infection, or infection of other organs [[7], [8], [9]].

*Nocardia pneumoniae* was first described by Kageyama et al., in 2004 [10]. Reviews on the clinical and laboratory features of *Nocardia* species do not assign definite clinical significance to *N. pneumoniae*, because the overall reported case number was below four [3,11].

Therefore, it is necessary to present additional cases to explore the clinical characteristics and therapy of *N. pneumoniae*. Here, we report on the first case of *N. pneumoniae* pulmonary infection in a patient with non-CF bronchiectasis in Europe and review previous *N. pneumoniae* cases.

## 2 Case presentation

A 54-year-old female with bronchiectasis of unknown etiology was referred by her pulmonary physician to our specialized bronchiectasis outpatient clinic in a tertiary German university hospital due to increased cough, sputum production and recurrent hemoptysis in the previous month. She did not respond to a three days course of azithromycin and ten days of cefpodoxime. Bronchiectasis severity index was 5, indicating moderate bronchiectasis [12].

The patient additionally experienced recurrent low-grade fever, weakness, and night sweats in the weeks prior to consultation. She had a one pack-year cumulative smoking history but stopped smoking over 20 years ago. The patient reported recurrent pulmonary infections since her childhood, which led to a right lung segmental resection approximately 40 years ago. Additional medical history included idiopathic epilepsy with generalized seizures since the age of 17. At the time of presentation, she had been seizure-free for the past seven years. The patient was on antiepileptic medication only, with no evidence of immunosuppressive medication. She reported no alcohol or drug use.

The family history was unremarkable for pulmonary disorders, including the patient's nine siblings and her 30-year-old son. However, the patient's parents are consanguineous (first degree cousins). The occupational history revealed no relevant exposures. The patient worked as an office clerk.

Initially, the patient presented with a slightly elevated C-reactive protein (0.57 mg/dl, norm <0.50 md/dl) and normal white blood cell count (WBC 6.44/nl (norm 3.96–10.41/nl)).

**Fig. 1:**
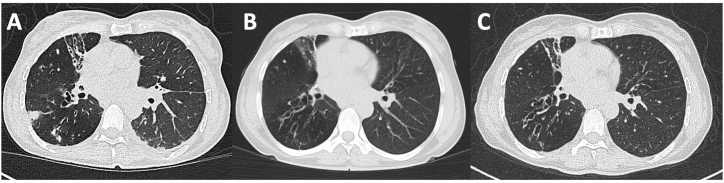
Axial thoracic CT images demonstrating bronchiectasis, mucus plugging, and extensive multifocal pulmonary consolidations (Fig. 1A). Follow-up CT scans after 8 weeks (B) and 12 weeks (C) of antibiotic therapy show bilateral regression of consolidations.

Thorax computed tomography (CT) confirmed bronchiectasis with a cranio-caudal gradient, mucus plugging and extensive multifocal lung consolidation without lymphadenopathy. Additionally, a small pleural effusion was detected on the left hemithorax (Fig. 1A).

Pulmonary function testing including spirometry and body plethysmography showed a moderate obstructive ventilatory disorder (FEV1: 1.03l, 40 %predicted). The diffusing capacity of the lung for carbon monoxide (DLCO) was reduced (67 %predicted), while the transfer coefficient (KCO = DLCO/VA) was within normal limits (104 %predicted), consistent with obstruction-related distribution disorder.

We collected a sputum specimen and bronchoscopy was performed. Endobronchial inspection revealed atrophic bronchitis with low-grade purulent secretions bilaterally. The orifice of the middle lobe bronchus showed narrowing secondary to strictures, consistent with prior surgery.

We found elevated total cell count (13.8 million, 0.63 million/ml) and marked increase in neutrophil count (94 % neutrophils, 4 % macrophages, 2 % lymphocytes) in the broncho-alveolar lavage fluid.

Microbiological cultures detected *Nocardia* sp. in both sputum and BAL samples, which was further classified as *Nocardia pneumoniae* using 16S rDNA sequencing.

Antimicrobial susceptibility testing (AST) was performed according to Clinical and Standards Institute (CLSI) recommendations by using MIC test stripes (Liofilchem, Italy). Based on the AST results (Table 1) a monotherapy with trimethoprim-sulfamethoxazole 800/160 mg three times per day was initiated.

**Table 1:** Antibiotic resistance profile.

antibiotic	MIC (μg/ml)	interpretation
ceftriaxone	0.25	sensitive
imipenem	0.5	sensitive
amikacin	0.5	sensitive
trimethoprim-sulfamethoxazole	0.032	sensitive
ciprofloxacin	>32	resistant
moxifloxacin	>32	intermediate
minocycline	1	sensitive
linezolid	1	sensitive

There was no hint for dissemination beyond the lungs based on cerebral MRI, transesophageal echocardiography, and abdominal ultrasonography.

Diagnostical workup was complemented by etiological assessment of the bronchiectasis. Alpha-1 antitrypsin deficiency was ruled out (already tested previously: 148 mg/dl, norm 90–200 mg/dl), IgG, IgA, IgM, and IgE levels were within normal limits. HIV-1/2 testing was negative. There was no evidence of *Aspergillus fumigatus* infection or allergic bronchopulmonary aspergillosis (ABPA). Rheumatoid factor was mildly elevated (20.9 U/ml; normal <14.0 U/ml), but anti-CCP antibodies were negative. Antinuclear antibodies (ANA) were borderline positive at a titer of 1:80; ANCA and ENA panels were negative. Cystic fibrosis was ruled out based on two independent and normal sweat chloride tests. IgG subclasses were within normal range. Nasal nitric oxide was pathologically decreased.

Genetic analysis was performed through WES (whole exome sequencing, NovaSeq Illumina, sequence analysis with SeqNext, JSI) with underlying gene panels for “primary ciliary dyskinesia” and “epilepsy” associated genes. No known pathogenic gene variant was identified.

In accordance with the diagnostic guidelines of the European Respiratory Society (ERS) and the American Thoracic Society (ATS), referral for ciliary ultrastructural analysis by transmission electron microscopy and high-speed video microscopy was offered [13,14]. However, the patient declined to undergo these investigations.

Although the bronchiectasis was initially classified as idiopathic, the patient's history of severe pulmonary infections and partial lung resection several decades earlier suggests a likely post-infectious origin.

Follow-up CT scans after 8 and 12-weeks of antibiotic therapy showed regressing pulmonary consolidations bilaterally (Fig. 1B/C). The patient's clinical condition improved significantly. She experienced markedly less coughing, no sputum production (thus no further sputum samples could be collected), had no hemoptysis or episodes of fever, and felt less fatigued, allowing her to return to a more active lifestyle. The treatment was well tolerated, no adverse drug reactions were reported.

Antibiotic treatment was given for 12 weeks, and a follow-up bronchoscopy was performed thereafter. Here, total cell count was reduced (1.86 million, 0.06 million/ml) and neutrophil count was reduced compared to initial BAL, but still increased (35 % neutrophils, 53 % macrophages, 3 % eosinophils, 9 % lymphocytes). Microbiological cultures did not detect *Nocardia*.

## 3 Review of previously described cases with *N. pneumoniae*

The species was first described by Kageyama et al. [10], who isolated *N. pneumoniae* from sputum of a 69-year-old male Japanese patient with a history of lung cancer, diabetes, emphysema, and radiation pneumonitis. Although an extensive taxonomic description was provided, no further details about the clinical course of the patient were given.

Betrán et al. [15] isolated *N. pneumoniae* in a 15-year-old female with cystic fibrosis. Clinical significance was declared as transient colonization as it was detected in no more than two cultures in six months and no clinical worsening was reported without antibiotic treatment.

Nakagoshi et al. [16] described a case of pulmonary nocardiosis by *N. pneumoniae* in a 69-year-old immune-competent woman. The patient presented with productive cough, with normal WBC and slightly elevated CRP (0.92 mg/dl). In CT Thorax a cavitary lung lesion in the left upper lobe, and multiple nodules and consolidations, mainly in the lingula were detected. The patient was treated with imipenem for 2 weeks based on antimicrobial susceptibility test results and then switched to oral faropenem treatment. Two months after treatment started, CT showed significant improvement in all lesions.

Clinical characteristics of all *N. pneumoniae* cases are reported in Table 2.

**Table 2:** Clinical parameters/characteristics of *N. pneumonia* cases reported in literature.

age	sex	lung disease	systemic immuno-deficiency	clinical significance	antibiotic treatment	treatment success	reference
69	male	emphysema, lung cancer, radiation pneumonitis	no	unknown	unknown	unknown	Kageyama et al. [10]
15	female	cystic fibrosis	no	transient colonization	no	–	Betrán et al. [15]
69	female	no	no	lung infection	imipenem; faropenem	yes	Nakagoshi et al. [16]
54	female	non-CF bronchiectasis	no	lung infection	trimethoprim-sulfamethoxazole	yes	presented here

## 4 Discussion

We here presented a case of a 54-year-old woman with bronchiectasis and infection with *N. pneumonia.* This case further substantiates previous reports of *N. pneumonia* infection. We suggest assigning *N. pneumonia* clinical significance. A good clinical, radiological, and microbiological response to treatment with trimethoprim-sulfamethoxazole was observed after 12 weeks. However, the management of nocardiosis remains challenging, as robust evidence is lacking regarding optimal treatment regimens (e.g. monotherapy or combination therapy), appropriate duration of therapy, and the necessity of secondary prophylaxis [8,9].

The presence of bronchiectasis in our patient aligns with previous findings, where bronchiectasis has been reported in 67–80 % of immunocompetent individuals with pulmonary nocardiosis [17].

To our knowledge, no disseminated infection with *N. pneumoniae* has been reported so far.

Interestingly, in the case presented here, a nontuberculous mycobacterium (NTM) was initially isolated from the sputum as well. As the species identified was *Mycobacterium gordonae* and it was not detected in subsequent cultures, we considered this finding as a contamination or innocent bystander rather than a clinically relevant pathogen. However, co-isolation with NTM has been reported in up to 29 % of immunocompetent patients with nocardiosis [5,17]. Whether this observation reflects a mechanistic interaction between these pathogens or merely results from their preference for a similar ecological niche remains elusive.

Non-cystic fibrosis bronchiectasis has received increasing attention, with studies such as EMBARC highlighting its significance and the role of bacterial infections in disease progression [18]. This case underscores the importance of thorough microbiological assessment - including for rare pathogens - in patients with bronchiectasis.

## CRediT authorship contribution statement

**Adrian Endres:** Writing – review & editing, Writing – original draft, Project administration, Methodology, Investigation, Formal analysis, Data curation, Conceptualization. **Maria Pilot:** Writing – review & editing, Writing – original draft, Project administration, Investigation. **Michael Hogardt:** Writing – review & editing, Writing – original draft, Supervision, Investigation, Conceptualization. **Sarah Westeppe:** Writing – review & editing, Investigation. **Birgit Zirn:** Writing – review & editing, Writing – original draft, Supervision, Investigation. **Gernot Rohde:** Writing – review & editing, Writing – original draft, Supervision, Project administration, Investigation, Funding acquisition, Conceptualization.

## Declaration of competing interest

The authors declare that they have no known competing financial interests or personal relationships that could have appeared to influence the work reported in this paper.
